# Rapid development of image analysis research tools: Bridging the gap between researcher and clinician with pyOsiriX

**DOI:** 10.1016/j.compbiomed.2015.12.002

**Published:** 2016-02-01

**Authors:** Matthew D. Blackledge, David J. Collins, Dow-Mu Koh, Martin O. Leach

**Affiliations:** CR-UK Cancer Imaging Centre, Radiotherapy and Imaging Division, The Institute of Cancer Research and The Royal Marsden NHS Foundation Trust, London, United Kingdom

**Keywords:** Medical imaging, Radiology, Dicom visualisation, Computed tomography, OsiriX, Python, Dicom management

## Abstract

We present pyOsiriX, a plugin built for the already popular dicom viewer OsiriX that provides users the ability to extend the functionality of OsiriX through simple Python scripts. This approach allows users to integrate the many cutting-edge scientific/image-processing libraries created for Python into a powerful DICOM visualisation package that is intuitive to use and already familiar to many clinical researchers. Using pyOsiriX we hope to bridge the apparent gap between basic imaging scientists and clinical practice in a research setting and thus accelerate the development of advanced clinical image processing. We provide arguments for the use of Python as a robust scripting language for incorporation into larger software solutions, outline the structure of pyOsiriX and how it may be used to extend the functionality of OsiriX, and we provide three case studies that exemplify its utility.

For our first case study we use pyOsiriX to provide a tool for smooth histogram display of voxel values within a user-defined region of interest (ROI) in OsiriX. We used a kernel density estimation (KDE) method available in Python using the scikit-learn library, where the total number of lines of Python code required to generate this tool was 22. Our second example presents a scheme for segmentation of the skeleton from CT datasets. We have demonstrated that good segmentation can be achieved for two example CT studies by using a combination of Python libraries including scikit-learn, scikit-image, SimpleITK and matplotlib. Furthermore, this segmentation method was incorporated into an automatic analysis of quantitative PET-CT in a patient with bone metastases from primary prostate cancer. This enabled repeatable statistical evaluation of PET uptake values for each lesion, before and after treatment, providing estaimes maximum and median standardised uptake values (SUV_max_ and SUV_med_ respectively). Following treatment we observed a reduction in lesion volume, SUV_max_ and SUV_med_ for all lesions, in agreement with a reduction in concurrent measures of serum prostate-specific antigen (PSA).

## Graphical abstract

**Figure:**
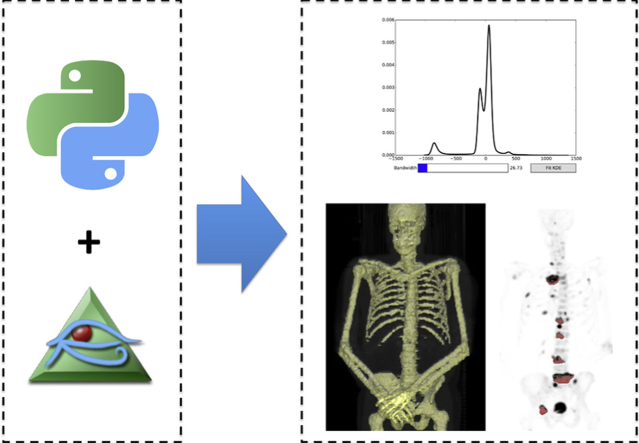

## 1 Introduction

A search for the keyword ‘Imaging’ in PubMed reveals that the number of medical imaging related articles published in 2014 exceeds 66,000 and that this number has been increasing over recent years. One major contributor is likely to be the increased utility of clinical and preclinical studies, which include magnetic resonance imaging (MRI), computed tomography (CT), diagnostic ultrasound (US) and positron emission tomography (PET) amongst others. Imaging provides a major advantage over other diagnostic techniques, as it is non-invasive and can provide in-vivo measurements of the biological properties of human tissue that can be monitored over time.

Imaging has revolutionized the standard for patient care and research in many fields of medicine including oncology [1], [2], [3], [4], [5], [6], [7], [8], [9], [10], cardiology [11], [12], [13], [14], [15], [16] and cognitive sciences [17], [18], [19], [20]. This increased reliance on imaging has evolved through technological innovations, enabling better diagnostic accuracy, faster acquisition times and lower costs. Furthermore, advances in computer hardware and storage now allow large patient datasets to be archived (e.g. up to several gigabytes per examination), which can be pipelined for advanced processing to enhance diagnostic performance. We predict that this trend will continue with further developments in multi-modal imaging [21], [22], [23], [24], [25], [26], quantitative imaging [10], [27], [28], [29], [30], [31] and radiomics/radiogenomics [32], [33], [34], [35], [36], [37], [38]. However, the rapid inflation in data volume necessitates the development of rapid and robust image analysis tools to ensure timely delivery of pertinent information.

The advent of the Digital Imaging and Communications in Medicine (DICOM) standard in the 1980’s has provided vendors with a common and lightweight means to store and distribute medical data [39], [40]. This revolution enables images acquired from different imaging modalities to be centrally accessed for patient diagnosis, and also to be searched and viewed on independent platforms. Most imaging systems now provide their own DICOM solution, which are also equipped with powerful visualisation tools such as multi-planar reformatting and volume rendering; alongside a comprehensive DICOM management framework. Furthermore, a number of independent alternative commercial DICOM visualisation platforms such as eFilm [41] and Olea Sphere [42] are also available.

An open-source DICOM imaging solution designed for the Apple® operating system, OsiriX, has gathered considerable interest amongst the medical community [43], [44], [45]. Built heavily using the Mac-native Objective-C programming language and libraries, it performs many of the vital medical image viewing tasks outlined above, including 2D/3D/4D data visualisation, DICOM database management and is able to act as a ‘Picture Archiving and Communication System’ (PACS) server for the retrieval of images transferred from other medical devices. It features an intuitive user-interface (UI) that is easy to use, which includes tools for defining Regions of Interest (ROIs) and basic image processing such as smoothing and segmentation. Of particular interest for imaging scientists is an open-source application program interface (API) that supports the development of custom-built ‘Plugins’ using the Objective-C programming language. At our institution, we have used this feature extensively to design and develop solutions for clinical research trials, where a number of novel image processing algorithms may be tested. These have included a plugin for computed diffusion weighted MR-imaging (cDWI) to improve the assessment of skeletal metastasis [27] and the calculation of fat-fraction from fat-water MRI techniques, amongst others. Although using the OsiriX API has provided our clinicians with useful clinical tools, this approach is impractical for research. The development of low-level C-based software is too cumbersome for rapid prototyping and lacks the scientific libraries available to scientific languages such as Matlab, IDL and Python. In this article we present our solution: pyOsiriX is a plugin that has been developed with the aim of providing researchers access to the data and functionality contained within OsiriX by means of using Python scripts. Using pyOsiriX accelerates the development of research tools for image processing and provides the availability of many advanced image processing algorithms through 3_^rd^_ party Python libraries: Numpy, Scipy, matplotlib, Scikit-Image, Scikit-Learn and the Insight Toolkit (ITK) to name but a few [46], [47], [48], [49], [50], [51]. Scripts developed using pyOsiriX can be made immediately available to clinicians for research within the familiar OsiriX user-interface, thus bridging the gap between image scientists and clinical practice.

## 2 Why Python?

Conceived in the late 1980’s, Python is rapidly gaining popularity as a high-level, open-source programming language amongst many software developers. It was designed with a vision to produce code that is readable and self-documenting, whilst retaining an object-oriented programming paradigm that is highly extensible. Python is a scripting language that relies on an interpreter to read and execute commands provided through terminal instructions or python files (defined by a ‘.py’ extension). Although the additional step of code interpretation can slow a program’s run performance when compared with pre-compiled machine-native code (written for example using C or C++), it is extremely advantageous for the research programmer. Important reasons for this are:

• Python interpretation eliminates the need to compile code every time it is to be run. From the perspective of OsiriX plugins, this means that plugins do not need to be reinstalled each time they are altered in debugging. This issue, in the view of the author, has been a major roadblock in the development of plugins, which require OsiriX to be restarted many times during development.
• All memory management is taken care of automatically by the interpreter. If a variable name is re-used, the old memory assigned to the variable reference is automatically deallocated.
• The interpreter is able to issue errors to the user without needing to stop the current runtime making the debug step of code development much simpler.
• As code written for interpretive languages can be saved as a text file, this automatically provides an easy way to store, audit and version-control algorithms in human readable format.

However, if execution time is an issue, Python provides a straight forward C API that makes it trivial to build Python libraries comprising native machine code to perform tasks at speed. The C API also provides the possibility of embedding Python as a scripting interpreter in another application, a feature that is not available to other scripting tools such as Matlab and IDL. Furthermore, Python is becoming very competitive as a scientific programming tool [52], [53] thanks to the advent of many free and open-source scientific libraries including efficient array manipulation using NumPy [46], scientific computing using SciPy [50], extensive plotting routines with matplotlib [48], advanced image processing with scikit-image [50], state-of-the-art machine learning using scikit-learn [49] and Bayesian modelling with PyMC [54]. Furthermore, Python builds of the Insight Segmentation and Registration Toolkit (ITK) [51] provide fast and easy access to some of the most advanced image segmentation and registration algorithms available to the imaging community. As a result of current developments we speculate that Python will be a major contender as the programming language of choice in scientific imaging research.

## 3 The structure of pyOsiriX

**Fig. 1:**
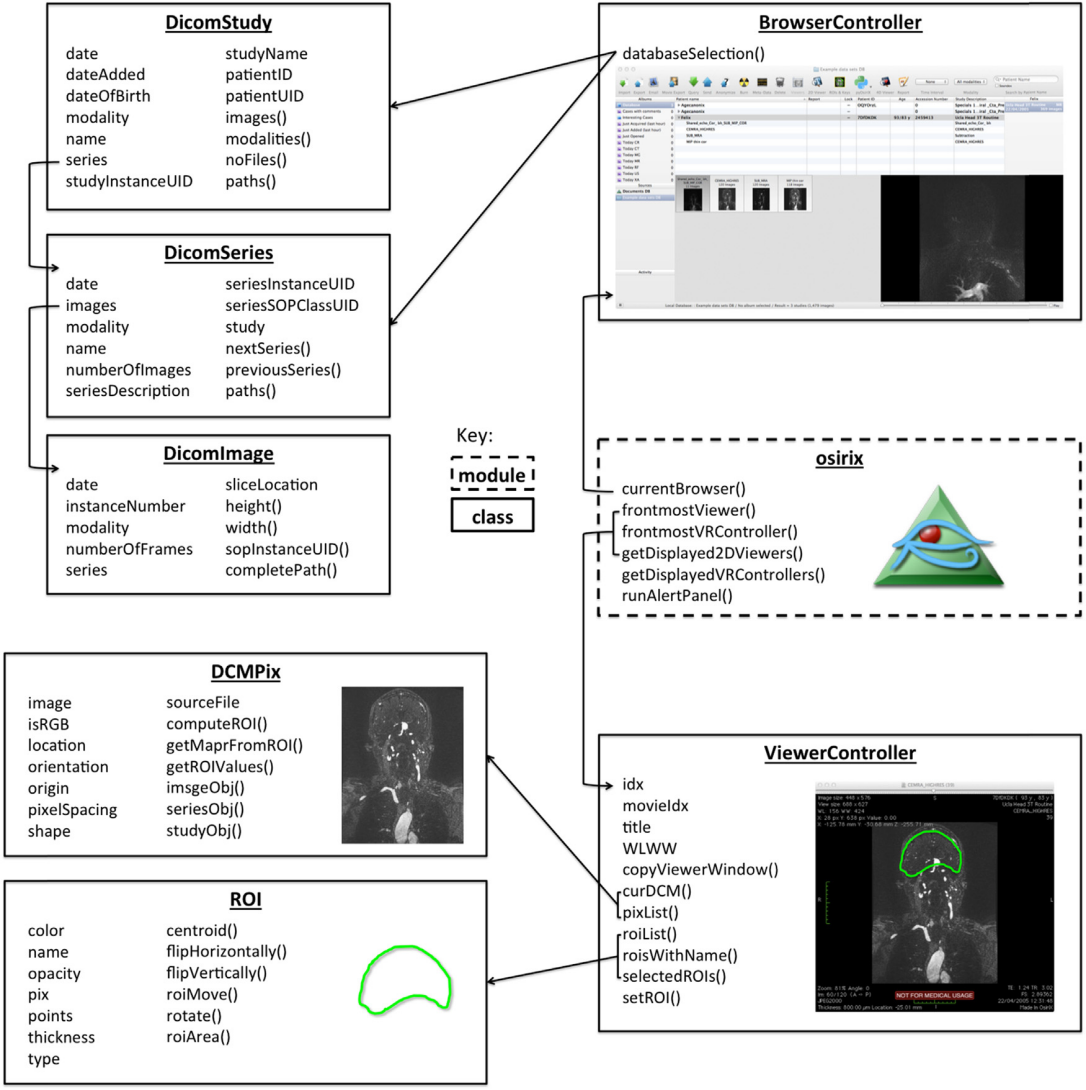
A diagram illustrating key elements of the pyOsiriX framework. Functions in the top-level module, ‘*osirix***’** (outlined by a dashed box), can be used to access various elements in OsiriX, which can in turn be used to explore the data more fully using existing scientific Python libraries.

As demonstrated in Fig. 1, pyOsiriX consists of a single Python module, ‘osirix’, and multiple classes that allow the user to manipulate the displayed images and regions of interest (ROIs), whilst also granting access to elements of the OsiriX database (using the DicomImage, DicomStudy and DicomSeries instances). With the exception of ROIs, new instances of each class are not allowed, as this is left to the OsiriX runtime. Rather, they are accessed using the naming conventions illustrated in Fig. 1. For example, if a user wishes to have access to the currently active 2D ViewerController (the primary 2D viewing window in OsiriX), the following Python syntax would suffice:

**Figure:**
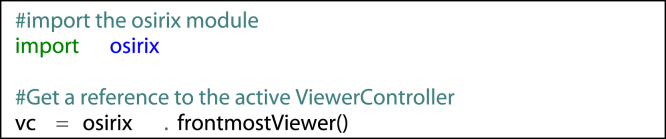

Each ViewerController provides access to lists of the contained DCMPix instances, which in turn are containers for Numpy arrays of pixel values and other relevant attributes such as the slice location, the dimensions of the contained image array and the location of the source file (from which DICOM attributes may be read using the Python dicom library pyDicom [55]). If a time series (e.g. obtained in a dynamic imaging study) has been loaded into the ViewerController (4D mode), the DCMPix list at a specific time frame is referenced as follows:

**Figure:**
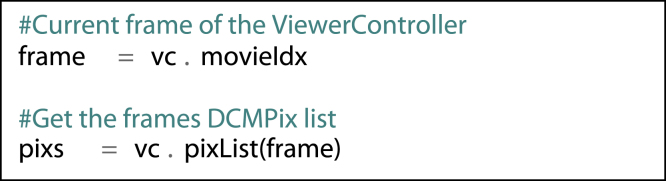

The Numpy array of pixel values for the currently displayed DCMPix may be accessed and manipulated for display:

**Figure:**
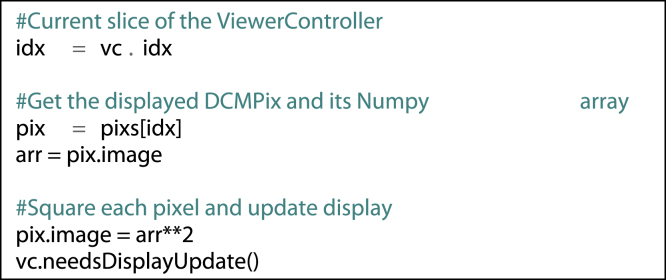

Within each ViewerController, also reside the lists of ROIs at each slice location (one list per movie frame). In order to access the currently displayed ROIs, the following code could be used

**Figure:**
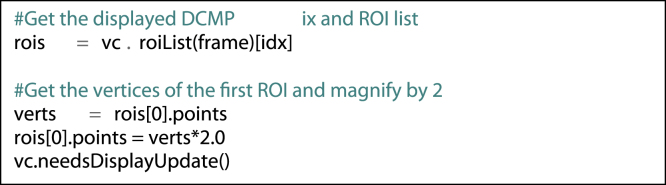

**Fig. 2:**
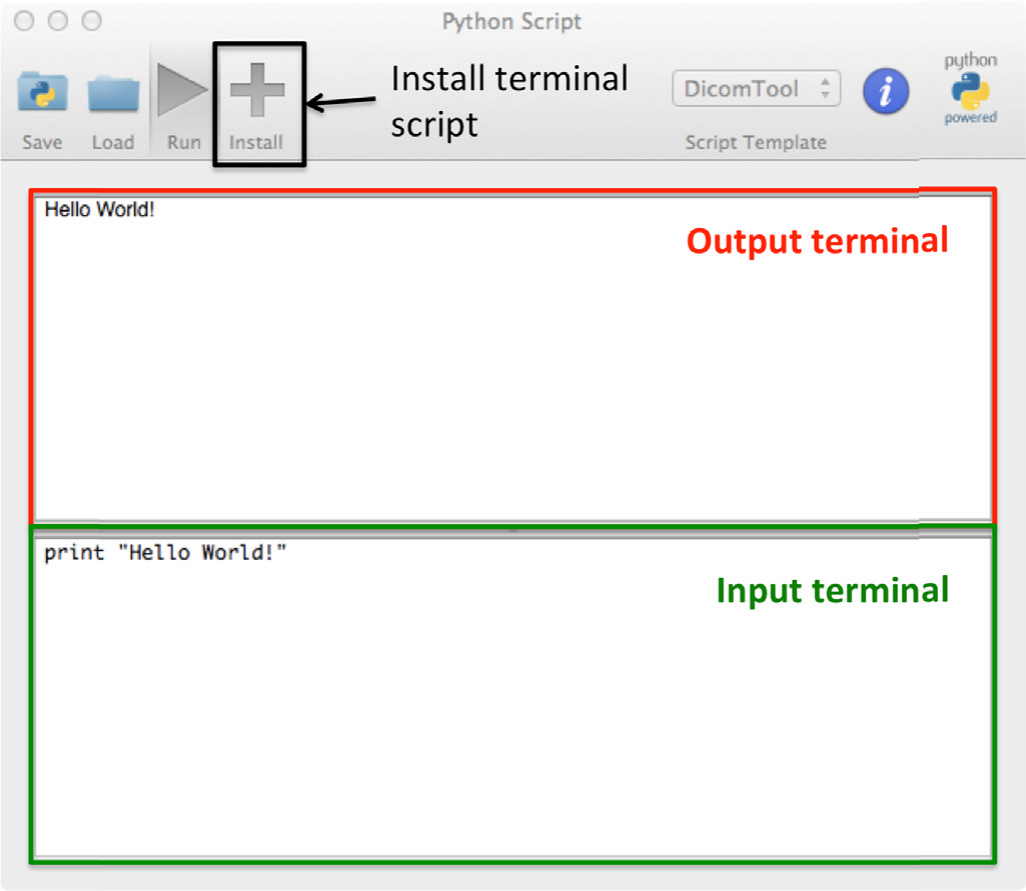
A screenshot of the pyOsiriX-scripting terminal. This plugin tool can be used to write and run scripts, provides script templates to aid script development and also enables the user to permanently install scripts so that the code remains hidden.

In designing pyOsiriX, we have ensured a naming convention as close to that of the underlying OsiriX source code as possible. In this way it is relatively straight forward for an engineer to translate any code written as Python scripts into fully native OsiriX plugins (Objective-C). Although the above exemplar statements are important for any scientific programmer wishing to use pyOsiriX, there are many other functions available, which will be fully documented at the application website [https://sites.google.com/site/pyosirix/]. Furthermore, by calling Python’s ‘help’ routine on any of the pyOsiriX classes and functions, the user is presented with documentation for the object in the form of a familiar Python ‘docstring’ displayed in the interactive terminal (Fig. 2).

## 4 pyOsiriX plugins

Python commands may be entered and executed via a simple interactive terminal (Fig. 2). This provides users with the ability to interact with code and the data displayed by OsiriX in real-time without the compilation steps necessary for native OsriX plugins, thus reducing programming complexity. Furthermore, if a script provides functionality that will be used repeatedly, it may be registered as a pyOsiriX plugin then executed by a button click. If scripts are to be used in this manner, the developer must provide header information at the top of the the python file as follows:

**Figure:**
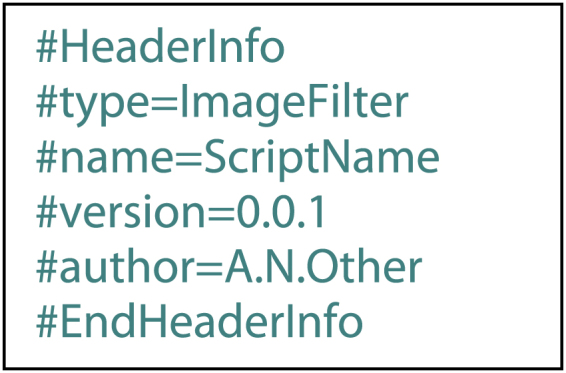

The ‘type’ field of the header determines under which group in which to display the plugin execution button, the ‘name’ provides the name of the execution button, and the ‘version’ and ‘author’ fields provide lightweight documentation for version-control. Currently supported ‘types’ include: ImageFilter, ROItool, DicomTool, DatabaseTool, VRtool and Other. Furthermore, when a plugin is installed, the results of a secure hash algorithm on the entire script (SHA-2) are stored on an Apple^®^ keychain so that modifications to the original source can be detected. This retains the open-source nature of the python script whilst ensuring that there are consistency checks for it to run.

## 5 Case studies

To demonstrate the utility of pyOsiriX we present three clinically motivated examples for analysis of X-ray computed tomography (CT) imaging and its combination with positron emission tomography (PET).

### 5.1 Kernel density estimation of ROI statistics

Our first example is relatively trivial in terms of its application, but it does highlight several important aspects of pyOsiriX that support its utility. The problem, simply stated, is as follows: Given several ROIs drawn in the currently displayed image of a ViewerController, plot the histogram of voxel values. Although, OsiriX does already provide a basic histogram tool, it lacks several features including: no *x*-axis or *y*-axis labels, no ability to change the plot colour and no functionality to save the resultant image. Furthermore, it uses the conventional number counting definition of a histogram by plotting along the y-axis the number of voxels whose values are between certain ranges. In-fact, this form of distribution may be considered a special case of Kernel Density Estimation (KDE), also know as Parzen windowing [56], [57]. Briefly, this technique is a non-parametric estimation method for determining the probability density function of a dataset through a normalised summation of ‘kernels’, *K*, with bandwidth *h* each centred about a single datum *x*_*i*_.

$$f_{h}\left(x\right)=\;\frac{1}{Nh}\sum_{i\;=1}^{N}K\left(\frac{x-\;x_{i}}{h}\right)$$

**Fig. 3:**
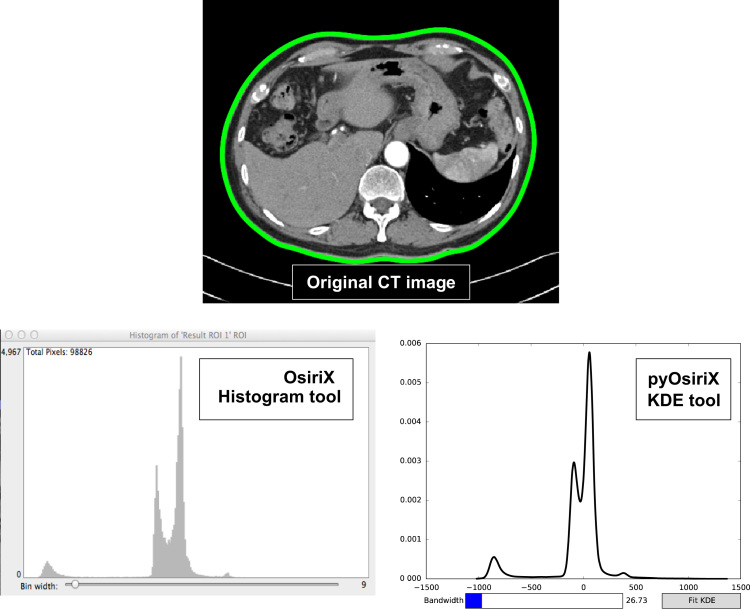
*Top**:*** an axial chest CT with a ROI (in green) drawn manually to outline the body. *Left*: a histogram of Hounsfield unit (HU) values generated using the in-built OsiriX histogram tool. *Right*: a kernel density plot of the sane data using the kernel density estimation tool written as a pyOsiriX script. It is clear that the KDE histogram can provide a much smoother interpretation of the probability density function for the CT data. The addition of a slider for choosing the bandwidth of the kernel density estimation provides the user with the ability to modify the level of histogram smoothing. (For interpretation of the references to color in this figure legend, the reader is referred to the web version of this article.)

A commonly used kernel is the Gaussian function, which has the advantage that the resulting PDF is smooth. Although this algorithm is straight forward to implement in principle, a full calculation is extremely inefficient and has O(*MN*) complexity where *N* is the number of data and *M* is the number of PDF positions that require calculation. A much more efficient implementation utilises the inherent sparsity of the data to turn the problem into O(*M*+*N*) time [58]. Thankfully, this functionality is already implemented in Python through the scikit-learn package [49]. We use this implementation along with several pyOsiriX methods and the matplotlib package to produce kernel density estimates of PDFs for a group of ROIs. The Python script for this functionality is provided as Supplementary material: KernelDensity.py. Note that the total number of lines of code needed to perform this task is 40 (not including comments), which could in principle be shortened were it not for the want of clarity. A demonstration of this tool is presented in Fig. 3, where it is compared to the result from the in-built histogram functionality of OsiriX using the PANORAMIX CT dataset provided on the OsiriX website [59]. It is clear that with relatively little written code, it is possible to produce very similar results to those provided by OsiriX. Moreover, the Kernel Density plot is smoother and the plot window displays the necessary axis values: CT values (Hounsfield Unit) along the *x*-axis and probability density along the *y*-axis. The widget functionality available in matplotlib [48] also prides the user with a simple interface that they may use to investigate different smoothing bandwidths. Furthermore, the resulting plot is interactive in the sense that the user is able to zoom into specific areas and also save an image of the results. This example made use of the matplotlib [48] and scikit-learn [49] packages, which are freely available and open-source.

#### 5.1.1 Segmentation of the axial skeleton in CT

**Fig. 4:**
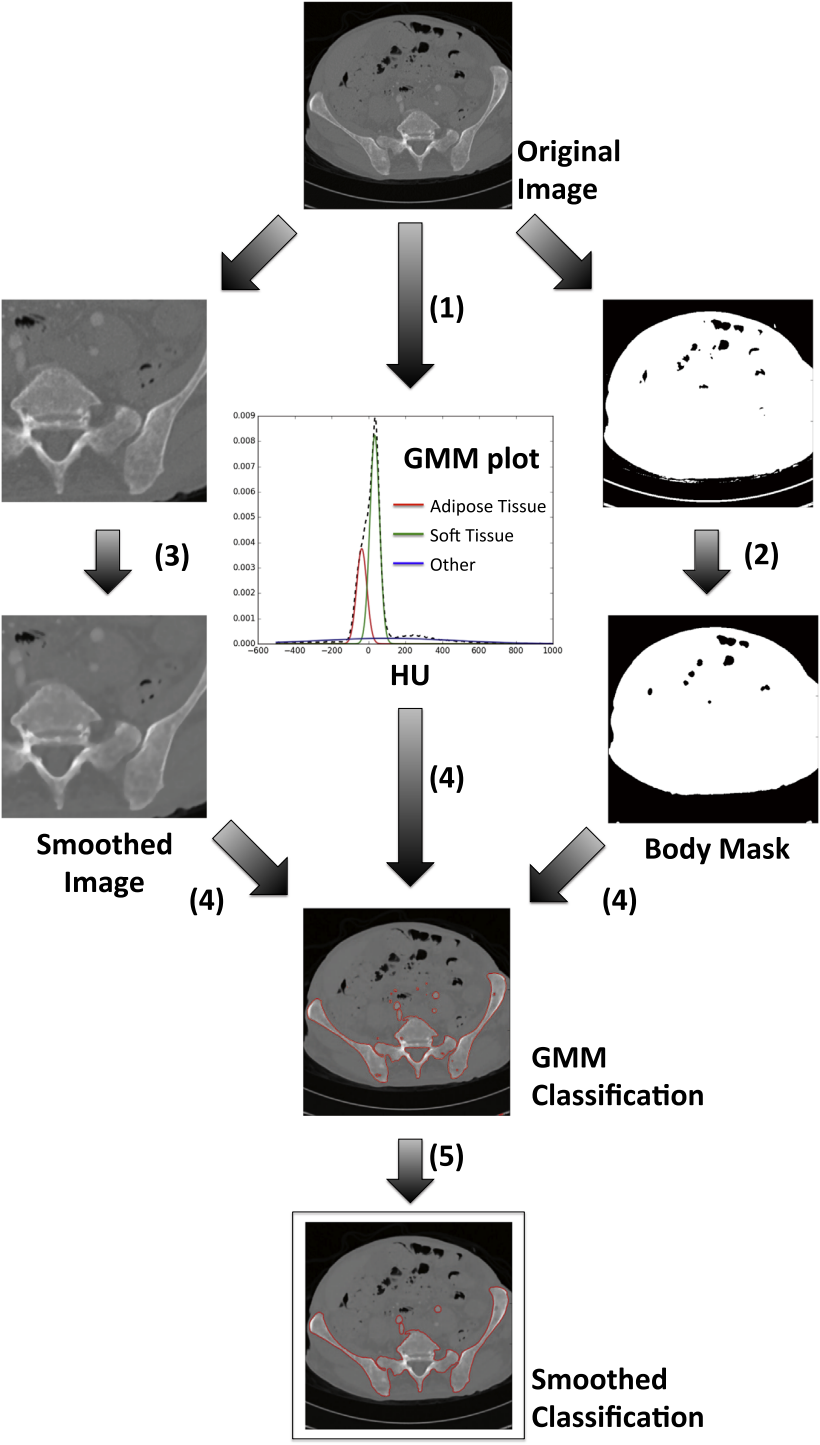
A workflow schematic demonstrating the image process steps used for a simple skeleton segmentation algorithm using CT data. A 3-class Gaussian Mixture Model (GMM) is fitted to the entre CT volume (1) and applied to a smoothed version of the images (3) using a morphologically smoothed body mask (2) to ensure the scanner bed is not included. The resulting segmentation (4) is improved by removing holes within the bone and removing regions less than a specified size (5).

In this example we segment the skeleton from whole-body CT datasets. Other authors have visited this well-known problem many times in the past and there are now a wide variety of techniques that provide a solution [60], [61], [62], [63]. Nonetheless, we demonstrate that good segmentation can be achieved using a combination of Python libraries with easy implementation in OsiriX for visualisation. By inspecting the example axial CT image shown in Fig. 3, it is observed that a number of imaging features must be accounted for when implementing a segmentation strategy for bone. Firstly, imaging noise (assumed to be Gaussian for large signal to noise ratio, SNR [64]) can result in a noisy segmentation result and so must be adequately modelled and accounted for in any algorithm. Second, CT imaging artefacts are visible as ‘streaking’ throughout the image and occur as a result of the reconstruction from under-sampled X-ray projections. The presence of any external objects, such as the scanner couch, pose a problem for any automatic segmentation schemes, and bone is composed of many tissue types including cortical bone, red and yellow marrows and vasculature, all of which will have varying CT values. Our proposed algorithm consists of a number of image filtering and segmentation steps that, in isolation, would be time consuming to develop from scratch in native Objective-C. However, by utilising general-purpose scientific Python libraries that already implement many of the required steps (referenced below), algorithm development is drastically reduced. Our approach, designed to cope with CT images acquired at different pixel resolutions, has a total 66 lines of Python code (ignoring all comments). Furthermore, we note that utility of Python libraries such as Numpy can vastly improves the efficiency of computation due to extensive optimisation during development [46]. Our algorithm is present below, where numbers correspond to those illustrated in Fig. 4. The full Python code is provided as Supplementary material SkeletalSegmentationCT.py.

1. *GMM Analysis*. From the results in Fig. 3, it is evident that the CT signal can be grouped into several distinct classes corresponding to different tissue types. Two peaks in the CT histogram are visibly centred on values of approximately from −100 to −50 (adipose tissue) and 0–100 (soft tissues). Furthermore, there is another peak around −700 that represents air, and is easily ignored by thresholding the image above -500. This histogram is modelled as a 3-class Gaussian Mixture Model (GMM) using the scikit-learn package by initialising Gaussian classes at – 100 (fat), 0 (water) and 500 (other) and then optimising the fit using the expectation maximisation algorithm [49].
2. *Body mask creation*. To ensure no external objects are included in the segmentation a mask of the patient body is created by thresholding the entire image above HU=−700 and then removing thin objects from the resulting mask through morphological opening and closing operations using the scikit-image package [50].
3. *Image smoothing***.** To reduce the effect of noise on the segmentation result an anisotropic-diffusion smoothing filter is applied to the images using the SimpleITK Python package [65]. Briefly, this algorithm applies a non-linear, shift-variant smoothing kernel to the image that retains sharp signal boundaries, whilst smoothing areas of consistent signal intensity. Two parameters that can be tuned in this algorithm are the conductance of the filter (a conductance of 0 allows no smoothing across any boundary) and the number of iterations. Here, these are set to 1.0 and 10 respectively.
4. *Bone classification.* Using the GMM trained from step 1 and the body mask created from step 2, the smoothed image from step 3 is classified into each of the three possible categories: ‘water’, ‘fat’ and ‘other’. Those pixels classified as ‘other’ and have HU>0 are attributed to be regions of bone.
5. *Segmentation improvements.* After bone classification, any holes contained entirely within the bone mask and smaller than 20 cm^2^ are filled and any independent region smaller than 0.5 cm^2^ are removed. This step is performed using the region labelling algorithm available to scikit-image.

**Fig. 5:**
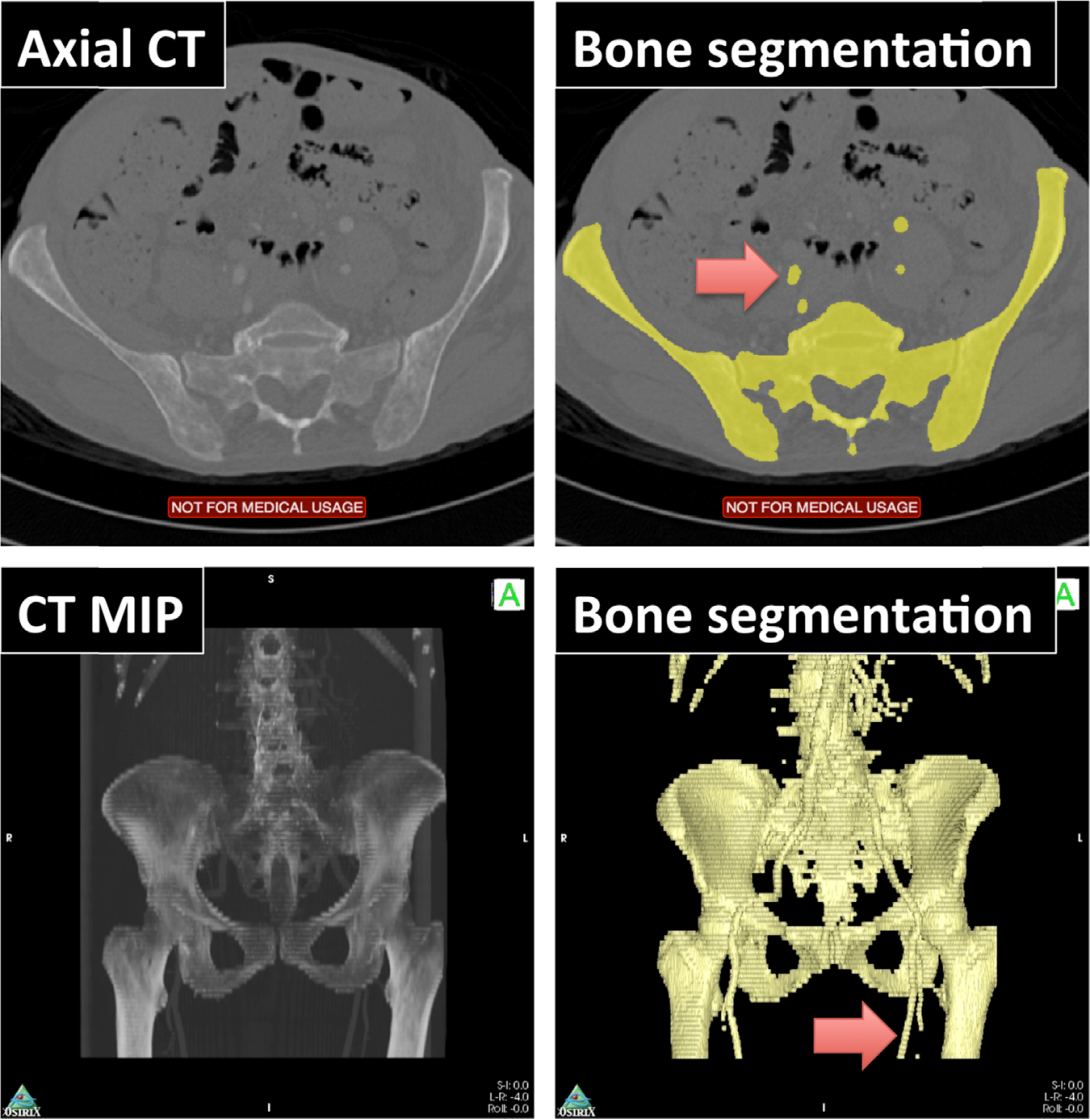
Automatic segmentation of CT angiogram in the pelvis. *Top:* segmentation results, displayed axially using a yellow brush ROI, demonstrate excellent delineation of the bone. However, the use of a CT contrast agent has also resulted in segmentation of some blood vessels (red arrow), which have similar CT density values to bones. *Bottom:* segmentation results shown as a 3D surface-rendered display (right) overlaid on a maximum intensity projection (left). (For interpretation of the references to color in this figure legend, the reader is referred to the web version of this article.)

**Fig. 6:**
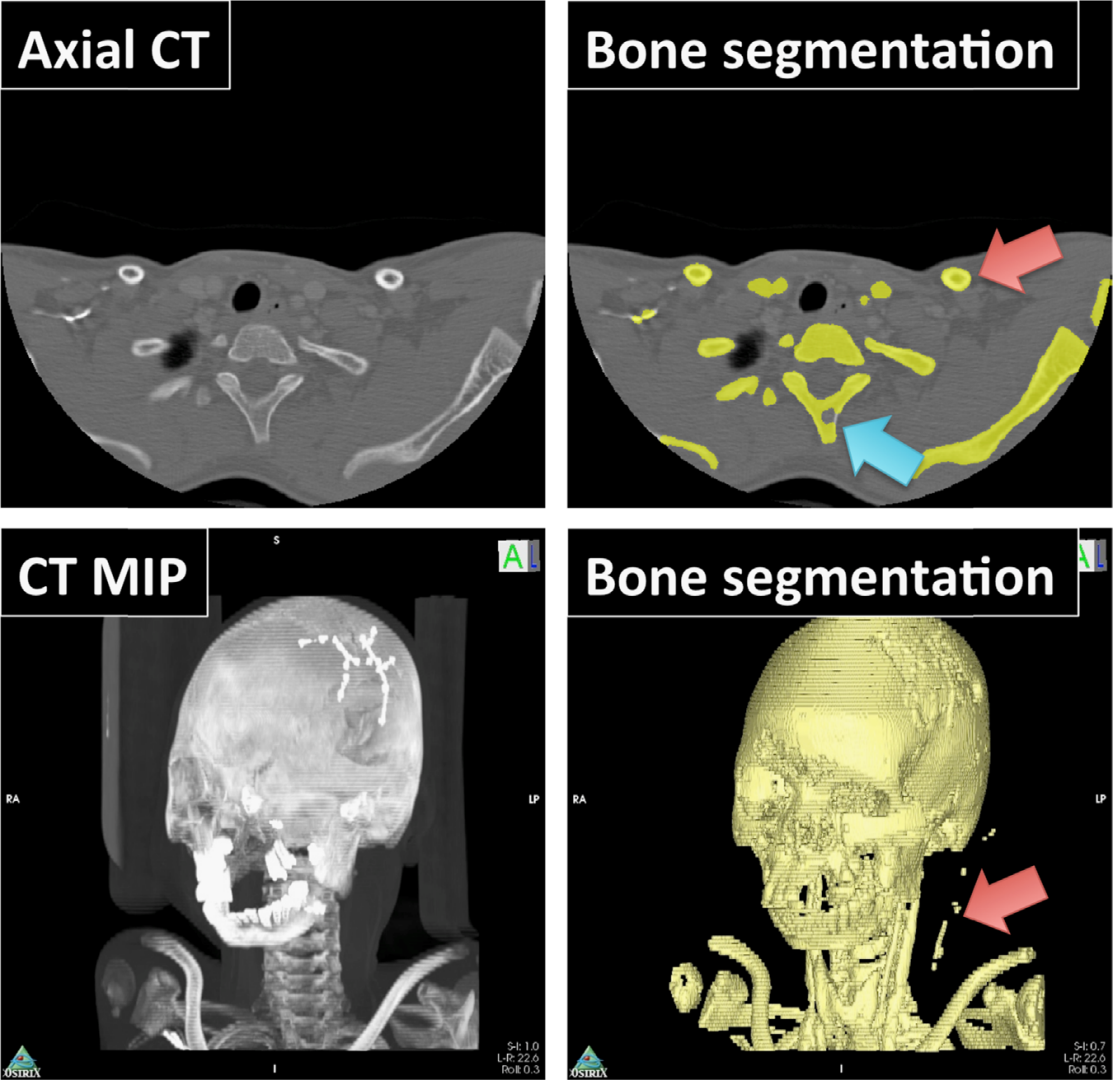
Automatic segmentation of Head CT dataset. *Top*: segmentation results, displayed axially using a yellow brush ROI, demonstrate excellent delineation of the bone. However, the use of a CT contrast agent has also caused segmentation of some blood vessels (red arrow). On occasion, imperfect bone segmentation was achieved (blue arrow). However, the clinician easily corrects for this using the ROI manipulation tools in OsiriX. *Bottom***:** Segmentation results shown as a 3D surface (right) overlain on a maximum intensity projection (left). (For interpretation of the references to color in this figure legend, the reader is referred to the web version of this article.)

This algorithm was also tested on the KESKRONIX and PHENIX CT angiography datasets available from the OsiriX website [59]. The results are presented in Fig. 5 and Fig. 6 respectively. Although both cases demonstrate inclusion of some larger blood vessels in the segmentation, it should be highlighted that both datasets are post contrast administration and so vessels appear hyperintense compared with standard CT. Otherwise, very good skeleton segmentation has been achieved in both cases. Performing such segmentation in OsiriX has the additional benefit that user modification of the resulting ROIs can be performed with ease using the many ROI modification tools already available to OsiriX. Furthermore, ROIs can easily be saved as a ‘.roi_series’ file and transferred from one study to another after image registration, thus facilitating multi-modal imaging analysis. Segmentation of these datasets took approximately 1–5 minutes (depending on input CT resolution) on a 1.7 GHz machine with 8 GB of RAM (MacBook Air).

#### 5.1.2 Segmentation of metastatic disease using ^18^F-fluoride PET-CT imaging

**Fig. 7:**
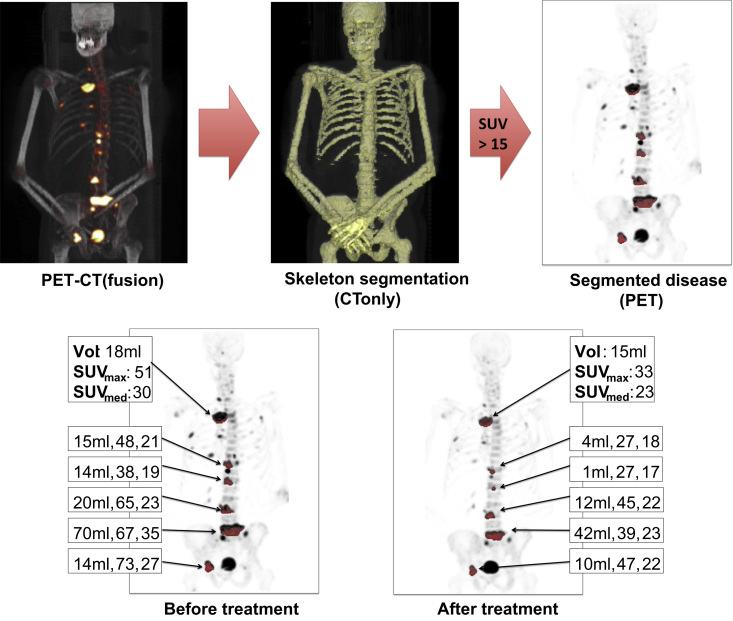
OsiriX provides an ideal environment to visualise multi-modal imaging datasets, such as in this example of an ^18^F-fluoride PET-CT study of a patient diagnosed with metastatic bone disease from prostate cancer. *Top***:** masks derived from skeletal segmentation of CT data (middle) is combined with a standardised uptake value threshold (SUV>15) to automatically define ROIs around regions of high fluoride uptake in the bone (right). *Bottom***:** using a region labelling algorithm of the final PET segmentation provides SUV statistics for each lesion before and after treatment. We report estimates of maximum and median SUV for each volume of interest (SUV_max_ and SUV_med_ respectively) and also estimates of lesion volume (Vol). A clear reduction in each of these parameters following treatment suggests patient response.

For our final case we demonstrate how pyOsiriX may be used to analyse multi-modal imaging datasets. ^18^F-fluoride was described as an agent for imaging bone over 50 years ago [66]. However, it is only recently that radiolabelled fluoride has gathered momentum as a reliable and sensitive tracer, largely due to the developments in positron emission tomography (PET) technologies and dual-modality PET-CT scanners [67], [68], [69], [70]. The application of ^18^F-PET-CT imaging to the assessment of metastatic prostate cancer is particularly attractive as response in this patient cohort is typically regarded to be non-measurable [71]. A recent study has demonstrated that the assessment of changes in the maximum standardised uptake value (SUV_max_) calculated within ROIs in ^18^F-PET-CT could provide a quantitative biomarker of disease response [68]. Our aim was to provide an automatic segmentation of suspect lesions on ^18^F-PET-CT, thus reducing the potential for measurement error from unrepeatable ROI definitions. Fig. 7 illustrates our segmentation scheme: Skeletal segmentation of the concurrent CT data was achieved using the algorithm described in the preceding section (CT images were resampled to the same voxel resolution as for PET using OsiriX). The intersection of the skeletal segmentation with a mask generated from thresholding the SUV images (SUV >15) provided the final PET segmentation; region labelling using scikit-learn provided separate ROIs for each spatially distinct lesion. Using this method we could automatically estimate changes in tumour volume, SUV_max_ and median SUV following treatment for each lesion independently. In an exemplar patient case (metastatic prostate cancer) we observed a reduction in all indices following treatment (Fig. 7), which was in agreement with a concurrent reduction in serum prostate-specific antigen (PSA) levels following therapy.

## 6 Discussion

In this article we present pyOsiriX, a simple yet powerful addition to the already popular OsiriX software package, allowing users to extend its functionality by using Python scripting. We envisage that pyOsiriX will quickly accelerate the transition of novel and relatively complex image processing algorithms from simple prototypes into robust image analysis tools for medical research. Clinical and scientific researchers will able to apply many cutting-edge scientific libraries that Python already provides to imaging data, and rapidly translate these for viewing using the many excellent tools available in OsiriX. In this article we have demonstrated three examples of the use of pyOsiriX in order to provide evidence that scripting the code of pyOsiriX can vastly reduce the number of steps required to perform complex image analysis tasks. In the first we generate kernel density estimates for histograms of voxel values contained within user-prescribed regions of interest (ROIs). This tool provides a smooth distribution with which the user may visually interrogate the number of classes that may be present in the image, which may then be related to the biological properties of the tissue evaluated. For our second example we demonstrate the utility of pyOsiriX for providing fast skeletal segmentation from body CT scans. By using existing Python libraries including scikit-learn, scikit-image, SimpleITK and matplotlib, we were able to rapidly implement an automatic CT segmentation scheme and volume render our results in OsiriX. Our process provided good segmentation of the bones on two example datasets freely available from the OsiriX website, with processing time on the order of one-two minutes depending on image resolution and field-of-view. Lastly, we have demonstrated that skeletal segmentation of CT can provide automatic delineation of suspect malignancies in ^18^F-radiolabelled fluoride PET-CT exams. This technique allowed us to export statistics of standardised uptake value (SUV) and lesion volume for each metastatic site: In an exemplar patient case with metastatic prostate cancer a reduction in mean SUV, median SUV and lesion volume was observed following treatment, in agreement with a concurrent reduction in serum PSA levels. All segmentation results were easily visualised using the excellent visualisation tools available in OsiriX, and could be managed using the OsiriX dicom database and stored on file.

We note that other powerful platforms for imaging research exist including MesVisLab [72] and 3DSlicer [73], [74]. However, we believe that the strength of pyOsiriX lies in its combination with OsiriX and bringing research tools onto an imaging viewing and analysis platform that is familiar to both imaging scientist and clinician. This will greatly facilitate collaboration between both disciplines and promote faster development of advanced clinical imaging methodologies.

We are undertaking a number of steps to facilitate and promote the development of pyOsiriX. First, we aim to ensure pyOsiriX as an open-source framework so that it can be supported and driven by the medical imaging community. Second, we wish pyOsiriX to be a stand-alone solution, and this will require integration of new open-source Python imaging libraries when they become available. Third, although a Python scripting terminal is provided, its current usage is basic and does not yet implement several scripting aids that can be useful for code development. In future we hope to include several debugging utilities such as breakpoints and function stepping routines.

Using pyOsiriX, we anticipate that clinicians and imaging scientists will be able to visually explore and interact with quantitative imaging data through the rich visualisation suite provided by OsiriX and develop robust image analysis tools using the many available Python libraries. We hope that these tools will bridge the apparent gap between these fields by incorporating fundamental imaging research into a framework and interface that is familiar to both. Ultimately we perceive that this will accelerate the development of advanced segmentation, registration and numerical analysis of medical imaging data into a robust clinical solution, supporting the application of techniques such as radiomics, radiogenomics and multi-modal, quantitative imaging.

## Grant support

We acknowledge CRUK and EPSRC support to the Cancer Imaging Centre at ICR and RMH in association with MRC & Dept. of Health C1060/A10334, C1060/A16464 and NHS funding to the NIHR Biomedicine Research Centre and the Clinical Research Facility in Imaging. Support also received from NIHR Postdoctoral Fellowship NHR011X. MOL is an NIHR Senior Investigator. The funders had no role in study design, data collection and analysis, decision to publish, or preparation of the manuscript. This paper presents independent research funded by the National Institute for Health Research (NIHR). The views expressed are those of the authors and not necessarily those of the NHS, the NIHR or the Department of Health.

## Role of funding source

CRUK & EPSRC - C1060/A10334, C1060/A16464 (MOL):

• Study design
• Manuscript preparation

NIHR - NHR011X (MDB):

• Study design
• Data analysis
• Development of analysis tools
• Manuscript authorship and preparation

## Footnotes

Appendix A Supplementary data associated with this article can be found in the online version at doi:10.1016/j.compbiomed.2015.12.002.
