# Supplementary material for: Rapid development of image analysis research tools: Bridging the gap between researcher and clinician with pyOsiriX
Source: Comput Biol Med. 2016 Feb 1;69:203–12. doi: 10.1016/j.compbiomed.2015.12.002 (PMC4761020; doi:10.1016/j.compbiomed.2015.12.002)
Supplement: Supplementary file 1 — Supplementary material [file mmc1.zip › SkeletalSegmentationCT_V2.rtf]

#HeaderInfo
#type=ImageFilter
#name=CT_SkeletalSegmentation
#version=0.0.1
#author=mblackledge
#EndHeaderInfo

#import the required libraries
import osirix
from skimage.morphology import closing, disk, convex_hull_image, opening, erosion, dilation, skeletonize
from skimage.measure import label
from sklearn.mixture import GMM
from SimpleITK import GetArrayFromImage, GetImageFromArray
from SimpleITK import GradientAnisotropicDiffusionImageFilter

#Create a vector of voxel data greater than a specified threshold
vc = osirix.frontmostViewer()
pixs = vc.pixList(0)
sh = pixs[0].shape
data = []
wait = vc.startWaitProgressWindow('Calculating data', len(pixs))
for j in range(len(pixs)):
	im = pixs[j].image
	data = np.r_[data, im[im > -700]]
	wait.incrementBy(1.0)
vc.endWaitWindow(wait)
#This MUST be called after a wait window is no longer needed!


#Create a Gaussian mixture model using 3 classes: fat, water, bone
#Initialise the
gmm = GMM(n_components = 3, init_params = 'w', params = 'wmc')
gmm.covars_ = np.reshape(np.array((1e3, 1e3, 1e6)), (3, 1))
gmm.means_ = np.reshape(np.array((-100., 0., 500.)), (3, 1))

#Fit the GMM on a random subset of the data
idx = np.floor(np.random.rand(100000)*data.size).astype('int')
data = data[idx]
gmm.fit(np.reshape(data, (data.size, 1)))

#Free some memory
del(data)

#Create an anisotropic diffusion smoothing filter
grad = GradientAnisotropicDiffusionImageFilter()
grad.SetNumberOfIterations(10)
grad.SetConductanceParameter(1.0)

#The following attemps to segment bone in each image
wait = vc.startWaitProgressWindow('Calculating bone mask', len(pixs))
boneMask = np.zeros([sh[0], sh[1], len(pixs)])
for j in range(len(pixs)):
	im = pixs[j].image
	pixelArea = np.prod(pixs[j].pixelSpacing)*0.01 #in cm^2

	#Create a mask of the body
	bodyMask = im > -300
	bodyMask = opening(bodyMask, disk(5))
	bodyMask = closing(bodyMask, disk(5))
	if bodyMask.max() == 0.0:
		continue

	#Smooth the image
	imSmoothed = GetArrayFromImage(grad.Execute(GetImageFromArray(im)))

	#Classify the image according to the GMM
	data = imSmoothed[bodyMask]
	cls = gmm.predict(data)

	#Create a bone mask for the image and smooth the result using morphological operations
	bone = np.zeros(sh)
	bone[np.where(bodyMask)] = cls
	bone = 1.0*(bone == 2)
	bone = closing(bone, disk(3))
	bone = opening(bone, disk(3))
	bone = bone/bone.max()

	#Remove any holes in the bone mask less than a specified area
	label_im, nb_labels = label(bone, return_num = True, connectivity = 2, background = 1.0)
	back_label = label_im[0, 0]
	for i in range(nb_labels):
		if (i+1 != back_label and np.sum(label_im == i+1)*pixelArea < 17.0):
			bone[label_im == i+1] = 1.0

	#Remove any unconnected regions smaller than a specified area
	label_im, nb_labels = label(bone, return_num = True, connectivity = 2, background = 0.0)
	for i in range(nb_labels):
		if ((np.sum(label_im == i+1))*pixelArea < 0.5):
			bone[label_im == i+1] = 0.0

	#Update the 3D mask and inform the user of progress
	mask[:, :, j] = bone
	wait.incrementBy(1.0)
vc.endWaitWindow(wait)

#Create new ROIs in each image to display the results
for j in range(len(pixs)):
	sl = mask[:, :, j]
	if np.sum(sl) > 0:
		m = sl/sl.max()
		roiNew = osirix.ROI(itype = 'tPlain', buffer = m > 0, name = 'Skeleton', DCMPix = pixs[j])
		roiNew.color = (255, 255, 0)
		roiNew.opacity = 0.5
		vc.setROI(roiNew, position = j)

del(mask) #Free some memory
vc.needsDisplayUpdate() #Tell OsiriX it needs to update
