# Supplementary material for: Rapid development of image analysis research tools: Bridging the gap between researcher and clinician with pyOsiriX
Source: Comput Biol Med. 2016 Feb 1;69:203–12. doi: 10.1016/j.compbiomed.2015.12.002 (PMC4761020; doi:10.1016/j.compbiomed.2015.12.002)
Supplement: Supplementary file 2 — Supplementary material [file mmc2.zip › KernelDensity_V2.rtf]

#HeaderInfo
#type=ROITool
#name=kernelDensityPlot
#version=0.0.1
#author=mblackledge
#EndHeaderInfo

# import all necessary libraries
import osirix
import numpy as np
import matplotlib.pyplot as pl
from matplotlib.widgets import Slider, Button
from sklearn.neighbors import KernelDensity

## A function returning Silverman's approximation for
## the bandwidth used in kernel density estimation
def silvermanApproximation(data):
	return np.power(4.0/(3.0*data.size), 0.2)*np.std(data)

# Obtain references to the displayed DCMPix and ROI objects
vc = osirix.frontmostViewer()
pixList = vc.pixList(vc.movieIdx)
roiList = vc.roiList(vc.movieIdx)
pix = pixList[vc.idx]
rois = roiList[vc.idx]

# Return a numpy array of all data contained within ROIs
data = []
for roi in rois:
	roiData, dataLocations = pix.getROIValues(roi)
	data = np.r_[data, roiData]

#A global variable to determine the currently displayed bandwidth for the KDE
#Initialized using Silverman's approximation
currentBandwidth = silvermanApproximation(data)

#Fit the KDE using the current bandwidth and display
def fitKDE(val):
    kd = KernelDensity(kernel = 'gaussian', bandwidth = currentBandwidth)
    kd.fit(np.reshape(data, (data.size, 1)))    
    pdf = kd.score_samples(np.reshape(xRange, (1000, 1)))
    axPlot.cla()
    axPlot.plot(xRange, np.exp(pdf), 'k-', lw = 3.0)
    pl.draw()

#Callback function for the 'Fit KDE' button
def sliderCallback(val):
    global currentBandwidth
    currentBandwidth = val

#Set up the matplotlib window to display a plotting axis, a slider axis 
#and a button axis
f = pl.figure()
axPlot = f.add_axes([0.1, 0.15, 0.8, 0.8])
axSlide = f.add_axes([0.2, 0.05, 0.4, 0.05])
axButton = f.add_axes([0.7, 0.05, 0.2, 0.05])
xRange = np.arange(data.min(), data.max(), (data.max() - data.min())/1000)

#The range of permitted bandwidths
bwMax = 0.1*(data.max() - data.min())
bwMin = 0.01*bwMax

#Create the user-controlled slider and 'Fit KDE' buttons and link to their 
#callback functions
slider = Slider(axSlide, 'Bandwidth', bwMin, bwMax, valinit = currentBandwidth)
slider.on_changed(sliderCallback)
fitButton = Button(axButton, 'Fit KDE')
fitButton.on_clicked(fitKDE)

#Fit and the first KDE and display the window
fitKDE(None)
pl.show()
